# *Calotropis procera* extract inhibits prostate cancer through regulation of autophagy

**DOI:** 10.1111/jcmm.18050

**Published:** 2024-02-23

**Authors:** Palak Singh, Bodhana Dhole, Jaganmoy Choudhury, Anannya Tuli, Deepak Pandey, Thirumurthy Velpandian, Surabhi Gupta, Pradeep Kumar Chaturvedi

**Affiliations:** ^1^ Department of Reproductive Biology All India Institute of Medical Sciences New Delhi India; ^2^ Department of Ocular Pharmacology Dr. Rajendra Prasad Centre for Ophthalmic Sciences, All India Institute of Medical Sciences New Delhi India

**Keywords:** apoptosis, autophagy, *Calotropis procera*, cell migration, cell proliferation, prostate cancer, reactive oxygen species

## Abstract

Current treatment options available for prostate cancer (PCa) patients have many adverse side effects and hence, new alternative therapies need to be explored. Anticancer potential of various phytochemicals derived from *Calotropis procera* has been studied in many cancers but no study has investigated the effect of leaf extract of *C. procera* on PCa cells. Hence, we investigated the effect of *C. procera* leaf extract (CPE) on cellular properties of androgen‐independent PC‐3 and androgen‐sensitive 22Rv1 cells. A hydroalcoholic extract of *C. procera* was prepared and MTT assay was performed to study the effect of CPE on viability of PCa cells. The effect of CPE on cell division ability, migration capability and reactive oxygen species (ROS) production was studied using colony formation assay, wound‐healing assay and 2′,7′‐dichlorodihydrofluorescein diacetate assay, respectively. Caspase activity assay and LDH assay were performed to study the involvement of apoptosis and necrosis in CPE‐mediated cell death. Protein levels of cell cycle, antioxidant, autophagy and apoptosis markers were measured by western blot. The composition of CPE was identified using untargeted LC–MS analysis. Results showed that CPE decreased the viability of both the PCa cells, PC‐3 and 22Rv1, in a dose‐ and time‐dependent manner. Also, CPE significantly inhibited the colony‐forming ability, migration and endogenous ROS production in both the cell lines. Furthermore, CPE significantly decreased NF‐κB protein levels and increased the protein levels of the cell cycle inhibitor p27. A significant increase in expression of autophagy markers was observed in CPE‐treated PC‐3 cells while autophagy markers were downregulated in 22Rv1 cells after CPE exposure. Hence, it can be concluded that CPE inhibits PCa cell viability possibly by regulating the autophagy pathway and/or altering the ROS levels. Thus, CPE can be explored as a possible alternative therapeutic agent for PCa.

## 1 INTRODUCTION

Prostate cancer (PCa) is in fourth position in terms of new incidences and eighth in terms of cancer‐related deaths in both sexes with a death rate of 3.8% worldwide. Amongst men, it is the second most common cancer with a global death rate of 6.8% as of 2020.^1^ Androgen deprivation therapy (ADT), surgery and radiation therapy are the commonly available primary treatments for PCa patients.^2^ However, conventional treatments have several adverse side effects including cardiotoxicity, musculoskeletal alterations and neurotoxicity.^3^, ^4^ To reduce the side effects, several new therapies such as immunotherapy, complementary and alternative medicines (CAM), vaccines and their combinations are being tested for their efficacy.^5^

Complementary medicines often include crude plant parts in the form of extracts, powder and tablets.^6^ These drugs are frequently being used along with surgery, radiotherapy or chemotherapy, either as a replacement of chemical drugs or act in a synergistic manner with other chemical drugs in order to increase the efficacy of the treatment with minimal side effects.^7^, ^8^ The bioactive compounds naturally found in these plants act against cancer cells by targeting various pro‐ and anti‐oncogenic molecules and signalling pathways.^9^ Therefore, the plant derivatives and their constituent bioactive compounds are being actively tested for their therapeutic potential.^8^, ^10^

The genus *Calotropis* belongs to Asclepiadaceae family and consists of only six species.^11^ *Calotropis procera* is also known as milkweed, swallowwort,^11^ madar and sodom apple.^12^ It is a tropical shrub found in parts of Africa, Asia and other tropics.^13^ In India, it is majorly found in wastelands of Assam, West Bengal, Rajasthan and Punjab.^12^ Different parts of this plant such as seeds, flower, roots, leaves and milky latex are reported to possess insecticidal, analgesic, anti‐ulcers, cytotoxic, anti‐bacterial, hepatoprotective and cardioprotective properties.^13^ Different phytochemicals in *C. procera* latex have shown anticancer potential in both human and animal models, specifically against breast, liver, glioblastoma, colon and PCa.^14^, ^15^ Recently the leaf extract of *C. procera* has been shown to exhibit anticancer potential against lung^16^ and breast cancer^17^, ^18^ but its role in PCa has not been explored till date. Thus, the current study investigated the anticancer activity of the leaf extract of *C. procera* on PCa cells in vitro and its underlying mechanism.

## 2 MATERIALS AND METHODS

### 2.1 Reagents

F‐12 K, RPMI‐1640 and KSFM media, fetal bovine serum (FBS), cell culture plates, MPER reagent, CyQUANT LDH Cytotoxicity Assay kit, BCA (Bicinchoninic acid) kit and EnzChek Caspase‐3 Activity Assay Kit were purchased from Thermo Fisher Scientific (Waltham, MA, USA). 3‐(4, 5‐Dimethylthiazol‐2‐yl)‐2, 5‐diphenyl tetrazolium bromide (MTT), doxorubicin hydrochloride, dimethyl sulfoxide (DMSO) and 2′,7′‐dichlorodihydrofluorescein diacetate (DCFDA) were purchased from Sigma Aldrich (St. Louis, MO, USA). Antibodies were purchased from Abcam (Cambridge, UK), Cell Signaling Technology (Danvers, Massachusetts, USA) and Santa Cruz Biotechnology (Dallas, Texas, USA).

### 2.2 Preparation of *C. procera* leaf extract

Plant of *C. procera* was identified by its flowers, and the leaves were washed and air dried for several days to remove moisture. The leaves were then ground to fine powder in liquid nitrogen. The powdered sample was weighed and methanol (Qualigens, India) was added to it in the ratio of 1:10 weight/volume. The methanolic mixture was shaken for 24 h followed by drying in rotary vacuum evaporator at 50°C. The pellet obtained was dissolved in 80% ethanol at a concentration of 80 mg/mL and stored at 4°C till further use.

### 2.3 Cell culture

All the cell lines, PC‐3, 22Rv1 and RWPE‐1, were purchased from the American Type Culture Collection (ATCC) and grown in a 37°C incubator with 5% CO_2_. Androgen‐independent PC‐3 cells were cultured in F‐12K medium while androgen‐sensitive 22Rv1 cell line was cultured in RPMI‐1640 medium, along with 10% FBS for both cell lines. The normal prostate epithelial cell line, RWPE‐1, was cultured in KSFM medium containing 5 ng/mL of recombinant human epidermal growth factor and 0.05 mg/mL of bovine pituitary extract.

### 2.4 Cell viability assay

MTT assay was used to determine the effect of *C. procera* leaf extract (CPE) on viability of PC‐3, 22Rv1 and RWPE‐1 cells.^19^ 5000 cells/0.2 mL/well were seeded in 96‐well culture plates. After 24 h of plating, they were treated with varying doses of CPE (25, 50, 100, 200 and 400 μg/mL) for different time points (24, 48 and 72 h). 0.5 mg/mL MTT solution was added to the wells after removing the culture medium, and cells were incubated for 4 h at 37°C, 5% CO_2_. The MTT solution was then discarded and formazan crystals formed were solubilized in DMSO for 2 h. Absorbance at 570 nm was measured using TECAN plate reader (Mannedorf, Switzerland). The absorbance at 570 nm is directly proportional to the number of viable cells. The percentage cell viability was then calculated as:

$$\begin{array}{c} \text{Percentage cell viability}\;\left(\mathrm{CV}\%\right)\\ =\left(\text{Absorbance of treated cells}/\text{Absorbance of control}\right)\times 100. \end{array}$$

### 2.5 Colony formation assay

To determine the colony formation ability of PCa cells on CPE treatment, colony formation assay was performed as previously described.^20^ Briefly, the cells were seeded in 6‐well plates at different cell densities for vehicle control (100, 250, 500 and 1000 cells/well) and treatment group (1000, 2000, 3000, 4000 and 5000 cells/well). After the cells adhered, they were treated with IC₅₀ concentration of CPE (26.5 μg/mL for PC‐3 and 20 μg/mL for 22Rv1) or vehicle control (complete media with ethanol) for 48 h. After 48 h of treatment, the wells were washed and complete fresh media was added. The media was changed after every 3–4 days. When colonies appeared in CPE‐treated wells (>50 cells/colony), the cells were fixed with formaldehyde and stained with crystal violet. The colonies were then visualized and counted under the microscope and plating efficiency (PE) and surviving fraction were calculated as:

$$\mathrm{PE}=\left(\mathrm{No}.\;\text{of colonies formed in control}/\mathrm{No}.\;\text{of cells seeded}\right).$$

$$\begin{array}{c} \text{Surviving fraction}=\left(\mathrm{No}.\;\text{of colonies formed after treatment}\right)/\\ \;\left(\mathrm{No}.\;\text{of cells seeded}\times \mathrm{PE}\right). \end{array}$$

### 2.6 Wound‐healing assay

To determine the effect of CPE on the migration of PCa cells, wound‐healing assay was performed. For the wound‐healing assay, 2.5 × 10^5^ cells/well were seeded for PC‐3 and 6 × 10^5^ cells/well were seeded for 22Rv1 cells in 24‐well plates. At 80%–90% cell confluency, a scratch was made passing through the middle region of the well, followed by treatment with either IC₅₀ concentration of CPE or vehicle (complete media with ethanol) taken as control. The width of the scratch was measured at 0 h and 48 h of the treatment. The % wound closure was calculated by the formula:

$\%\text{Wound closure}=\frac{\text{Width}\;\mathrm{at}\;0\;h–\text{Width}\;\mathrm{at}\;48\;h}{\text{Width}\;\mathrm{at}\;0\;h}\times 100$.

### 2.7 Measurement of reactive oxygen species

The endogenous reactive oxygen species (ROS) levels on CPE treatment, were measured using DCFDA assay. For this assay, 5000 cells/well were seeded in 96‐well plates. Cells were treated with either IC₅₀ concentration of CPE or 500 μM Hydrogen peroxide (H₂O₂) (taken as positive control) for 48 h. After 48 h of treatment, 10 μM of DCFDA was added to each well for 1 h and fluorescence was measured using Tecan multimode microplate reader with excitation and emission at 488 and 525 nm respectively.

### 2.8 Measurement of necrotic potential

Necrotic potential of CPE on PCa cells was measured using CyQUANT LDH Cytotoxicity Assay kit as per the manufacturer's instruction. Briefly, 5000 cells/well were seeded in 96‐well plate and incubated for 24 h. After 24 h of incubation, treatment of CPE was given in designated wells and 10 μL of ultrapure water was put in cells for measurement of spontaneous LDH activity. The plate was incubated at 37°C with 5% CO₂ for 48 h. After the designated time period of treatment, media from the wells serving as the maximum LDH activity controls was replaced with fresh complete media containing 10 μL of 10× Lysis Buffer. The plate was then incubated at 37°C with 5% CO₂ for 45 min. 50 μL of medium from each sample (spontaneous LDH activity, maximum LDH activity and treatment LDH activity) was transferred to another 96‐well flat‐bottom plate and 50 μL of Reaction Mixture was then added to each sample well and mixed well. The plate was incubated at room temperature for 30 min protected from light. 50 μL of Stop Solution was then added to each sample well, followed by absorbance measurement at 490 nm and 680 nm using TECAN multimode microplate reader. The % Cytotoxicity was determined using the formula:

$$\begin{array}{c} \%\text{Cytotoxicity}=\left(\text{Treatment}\;\mathrm{LDH}\;\text{activity}–\text{Spontaneous}\;\mathrm{LDH}\;\text{activity}\right)/\\ \left(\text{Maximum}\;\mathrm{LDH}\;\text{activity}–\text{Spontaneous}\;\mathrm{LDH}\;\text{activity}\right)\times 100. \end{array}$$

### 2.9 Measurement of apoptotic potential

Caspase‐3 activity‐mediated apoptosis of CPE was measured using the EnzChek Caspase‐3 Activity Assay Kit, as per the manufacturer's instruction. 2 × 10^5^ cells were plated in a 24‐well plate in complete media (media with 10% FBS). After 24 h of seeding for PC‐3 (48 h for 22Rv1 cells), cells were treated with IC₅₀ values of the CPE for 48 h. H₂O₂ at a concentration of 50 μM was used as a positive inducer of caspase 3 activity. Post‐treatment, the cells were washed with 1 mL PBS per well and 60 μL of 1× lysis buffer was added to each well with the cells and kept on ice for 30 min. The lysed cells were then centrifuged for 5 min at 5000 rpm and 50 μL of each sample supernatant was added to 50 μL Z‐DEVD‐AMC substrate solution in a 96‐well black plate. The cells were then incubated at room temperature for 60 min and fluorescence reading was taken at excitation 342 nm and emission 441 nm using TECAN multimode microplate reader. A standard curve was prepared using a series of 7‐amino‐4‐methylcoumarin (AMC) solutions of known concentration ranging from 100 to 0.05 μM. The standard curve was used to calculate the amount of AMC produced in each sample. The caspase 3 activity in nmole of AMC released per min per mL of cell lysate or positive control was calculated based on the formula:

$$\text{Caspase}\;3\;\text{activity}\;\left(\text{nmol}\;\mathrm{AMC}/\min/\mathrm{mL}\right)=\left(\text{nmol}\;\mathrm{AMC}\times d\right)/\left(t\times v\right),$$

where v = volume of sample in mL; *d* = dilution factor; *t* = reaction time in minutes.

### 2.10 Western blot

The whole cell lysate from cells treated with CPE or vehicle control was isolated using MPER reagent along with inhibitors (1% phosphatase inhibitor, 1% protease inhibitor and 1% PMSF). BCA Assay was performed for measurement of protein concentration in the cell lysates. Equal amount of protein sample from vehicle control and CPE‐treated cells was separated using 12% or 15% SDS‐PAGE and then electrotransferred on to a PVDF membrane. The membranes were incubated (at 4°C) overnight, with primary antibody specific for NF‐κB, oxidative stress markers, LC3B, Beclin‐1, p62, BAD, Bcl‐2 (Abcam), BAX, p27 (Cell Signaling Technology) or GAPDH (Santa Cruz Biotechnology), followed by corresponding HRP conjugated anti‐rabbit IgG antibody (Cell Signaling Technology) or anti‐mouse IgG antibody (Cell Signaling Technology) incubation for 2 h. After that, the membranes were thoroughly washed in TBST (1×). Finally, the PVDF membrane was probed with ECL solution (Thermo Fisher) and observed in ChemiDoc Imaging System (Azure Biosystems) to visualize the protein bands.

### 2.11 LC–MS analysis

Liquid chromatography–mass spectrometry (LC–MS) analysis of the leaf extract of *C. procera* was performed using a high‐performance liquid chromatography system (HPLC; Agilent Technologies, Santa Clara, CA, USA) hyphenated to a triple quadrupole tandem mass spectrometer (4000 Q‐Trap, AB Sciex, Foster City, CA, USA). All the parameters of mass spectrometer and HPLC were controlled by Analyst software, version 1.7.1 (AB Sciex) and OpenLAB control panel software (Agilent Technologies), respectively. The crude extract was diluted to 1 mg/mL in pure methanol and further diluted to 10 μg/mL in 50% methanol containing 0.1% formic acid (FA) for LC–MS analysis. The chromatographic separation was carried out on a Purospher® STAR RP‐18 endcapped (3 μm) column using a mobile phase combination of solvent A (18.2 MΩ Milli‐Q water with 0.1% FA) and solvent B (pure methanol with 0.1% FA) pumped at a flow rate of 0.5 mL/min. The gradient profile was set as: 0.0 min 5% B eluent, 1.0 min 5% B eluent, 15.0 min 100% B eluent, 19.0 min 100% B eluent, 20.0 min 5% B eluent and 25.0 min 5% B eluent. The temperature of autosampler tray and the column oven were maintained at 10 ± 1 and 25 ± 1°C, respectively, and the samples were injected at a volume of 20 μL for analysis. In order to achieve maximum coverage of the components, ESI source was operated in dual ionization mode with polarity switching. Independent data acquisition using enhanced mass scan (EMS) mode in the range of 50–800 (m/z) was used for capturing the [M+H^+^] and [M‐H^+^] masses. The source‐dependent parameters were set as: curtain gas (CUR) = 25 psi, collision gas (CAD) = 12 psi, ion‐spray voltage = 4.5 kV and 5.5 kV for positive and negative mode respectively, source temperature = 500°C, ion source gas 1 (GS1) = 45 psi, ion source gas 2 (GS2) = 60 psi.

### 2.12 Identification of intracellular bioactive compounds

In order to analyse the CPE metabolites entering the cells, the PC‐3 and 22Rv1 cells were treated with IC₅₀ concentration of CPE. The cells were trypsinized after 24 h of treatment to give maximum time for the metabolites to enter the cells but not enough time for them to cause cell death. After trypsinization of treated cells, the cell pellet was washed with 1× PBS and centrifuged at 13,000×*g* for 20 min at 4°C, so as to form a tight pellet. The cell pellet was resuspended in 1 mL of nuclease free water. The cells were then lyophilized. The lyophilized cell lysates were reconstituted in 50% MeOH containing 0.1% FA. The resulting solution was vortexed for 1 min and centrifuged at 10,000 rpm for 10 min at 4°C, and the supernatant was subjected for LC–MS analysis as described above.

### 2.13 Statistical analysis

Data was analysed using GraphPad Prism, version 8.0 (GraphPad Software Inc). The dose‐response curve and IC₅₀ values for each cell line were calculated by non‐linear regression analysis. Mean ± SEM was calculated for three independent experiments, each with at least three technical replicates. Two‐way anova was used to determine statistical significance for all MTT assays, and unpaired *t*‐test with Welch's correction was used to determine the statistical significance for the results of all other assays and western blots.

## 3 RESULTS

### 3.1 Effect of CPE on cell viability

**FIGURE 1:**
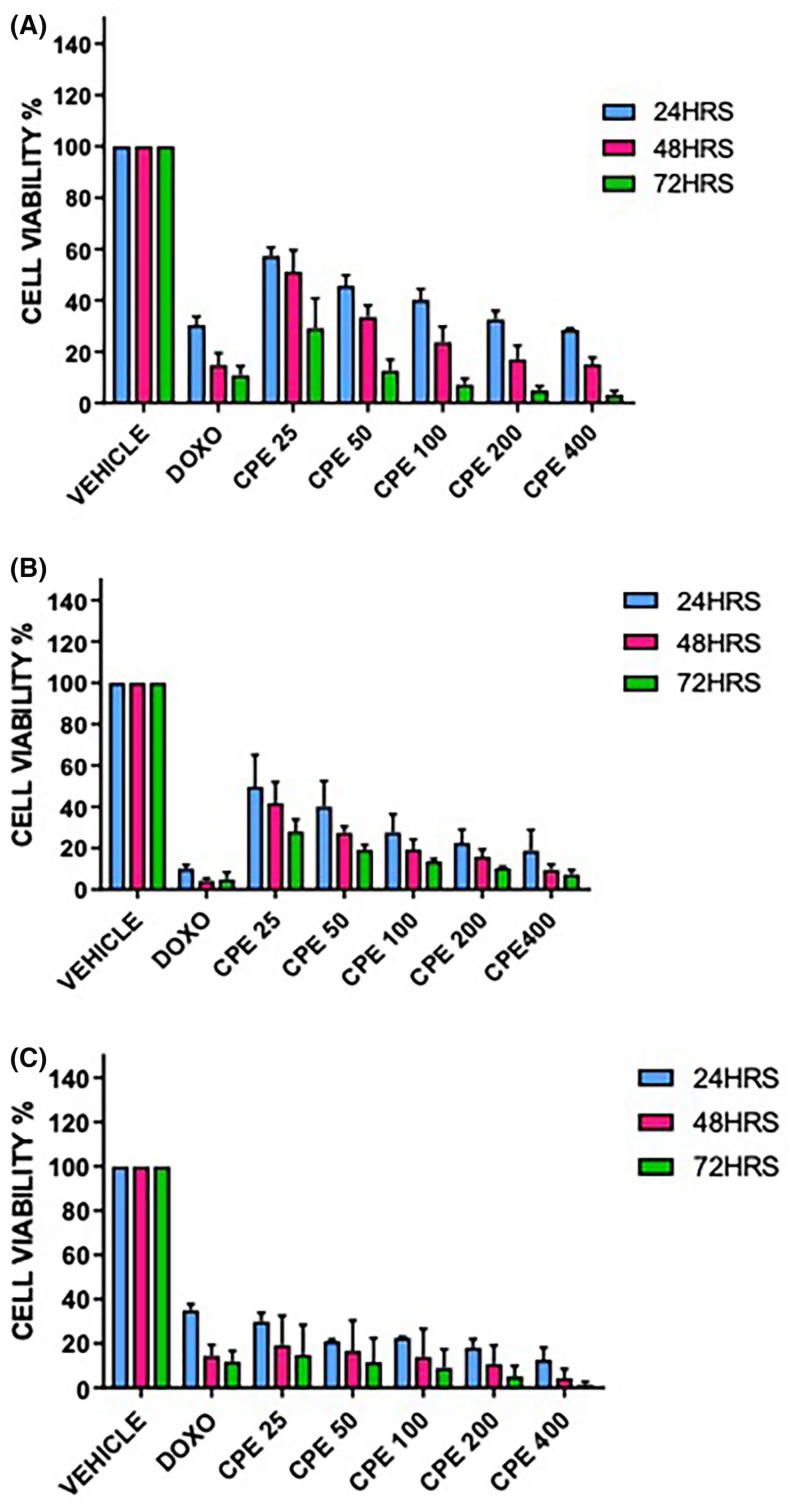
Effect of *Calotropis procera* leaf extract (CPE) on cell viability: Per cent cell viability at different time points (24, 48, 72 h) after treatment with varying concentrations of CPE (25–400 μg/mL). (A) PC‐3 cell line (B) 22Rv1 cell line (C) RWPE‐1 cell line. Data are presented as mean ± SEM (*n* = 3).

The effect of CPE on viability of human PCa cells, PC‐3 and 22Rv1, was measured by the MTT assay (Figure 1A,B). To measure the effect of CPE on viability of non‐cancerous cells, normal prostate epithelial cell line (RWPE‐1) was used (Figure 1C). Doxorubicin at a concentration of 100 μM was used as a positive control because it is one of the widely used chemotherapeutic drug for PCa. CPE decreased cell viability of PC‐3 and 22Rv1 cells in a dose‐ and time‐dependent manner. The IC₅₀ values of CPE for all the three cell lines (PC‐3, 22Rv1 and RWPE‐1) are given in Table 1. The optimal cytotoxic effect of CPE was observed at 48 h in both PC‐3 and 22Rv1 cell lines. Therefore, the PCa cells were treated with IC₅₀ concentration of CPE for 48 h in all further experiments.

**TABLE 1:** IC₅₀ concentrations of *Calotropis procera* leaf extract at different time points.

	IC₅₀ of CPE (μg/mL)		
	24 h	48 h	72 h
PC‐3	52.20	26.50	12.20
22Rv1	34.58	19.87	12.98
RWPE‐1	14.31	10.02	9.77

### 3.2 Effects of CPE on cell division ability of PCa cells

**FIGURE 2:**
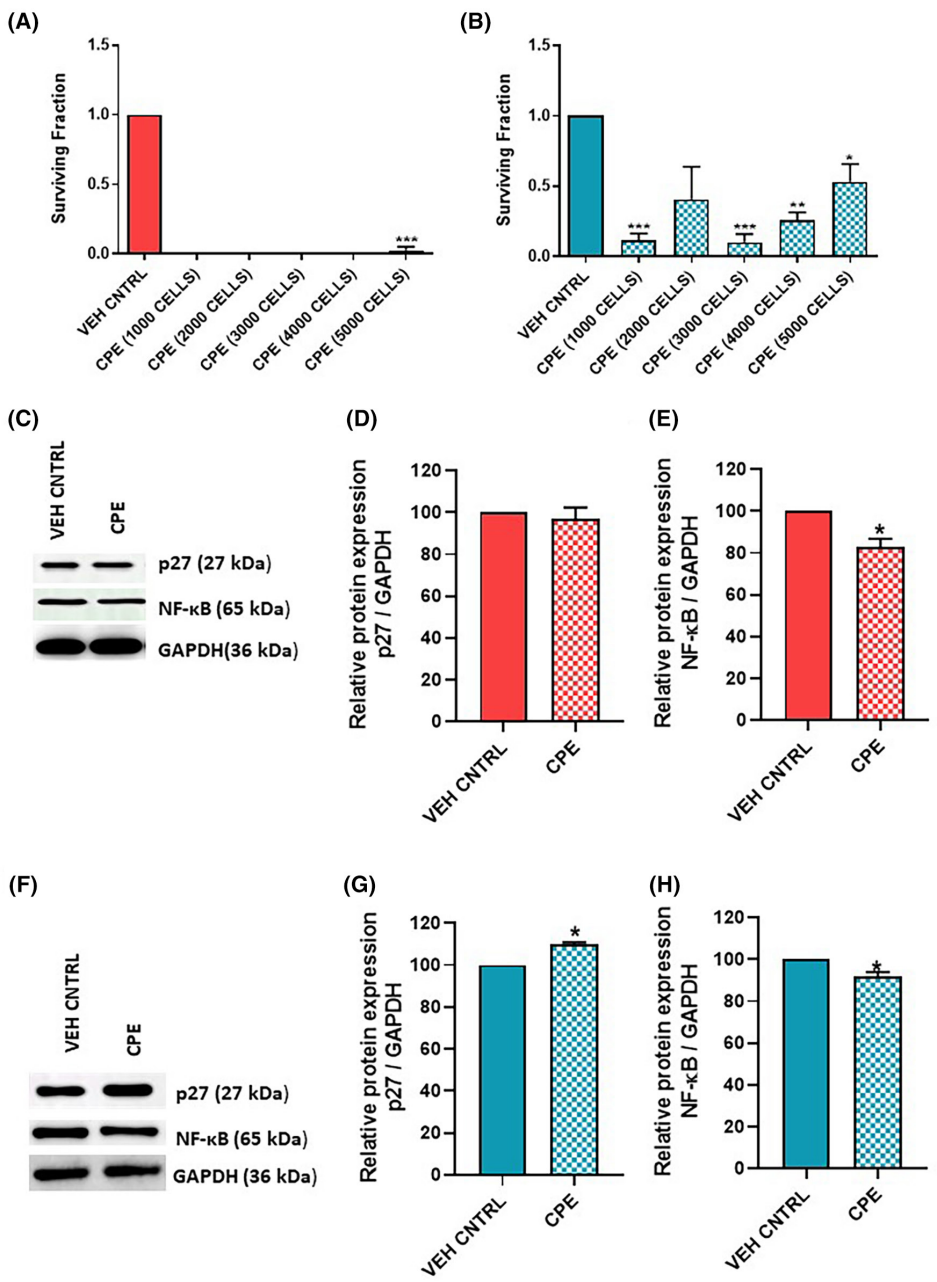
Effect of *Calotropis procera* leaf extract (CPE) on cell division ability measured via clonogenic assay and western blot of cell cycle markers in prostate cancer cell lines. (A, B) Histogram represents the survival fraction of the colonies formed in (A) PC‐3 and (B) 22Rv1 cells treated with CPE or vehicle control. (C–E) Representative western blot and densitometric analysis of cell cycle markers p27 and NF‐κB in PC‐3 cells. (F–H) Representative western blot and densitometric analysis of cell cycle markers p27 and NF‐κB in 22Rv1 cells. Data are represented as mean ± SEM (*n* = 3). *p < 0.05, **p < 0.01, ***p < 0.001 compared with vehicle control.

To determine the division ability of a single cell to form a colony after treatment with CPE, clonogenic assay was performed. The size and number of colonies formed in each cell line decreased after treatment with CPE as compared to the control. CPE treatment had a more profound effect on cell division capability of PC‐3 cells compared with 22Rv1 cells (Figure 2A,B).

### 3.3 Effect of CPE on cell cycle markers

Since a significant decrease in clonogenic ability of CPE‐treated cells was observed in both PCa cells, the protein levels of cell cycle markers, p27 and NF‐κB, were checked in both PC‐3 and 22Rv1 cells after treatment with CPE (Figure 2C,F). Densitometric analysis of the western blots showed no change in expression of p27 whereas a significant decrease in protein levels of NF‐κB (*p* ≤ 0.05) was observed in CPE‐treated PC‐3 cells (Figure 2D,E). On the contrary, an increase in p27 expression (*p* ≤ 0.05) was observed in CPE‐treated 22Rv1 cells as compared to vehicle control (Figure 2G). Furthermore, NF‐κB expression remained downregulated (*p* ≤ 0.05), in CPE‐treated 22Rv1 cells as well (Figure 2H).

### 3.4 Effect of CPE on cell migration

**FIGURE 3:**
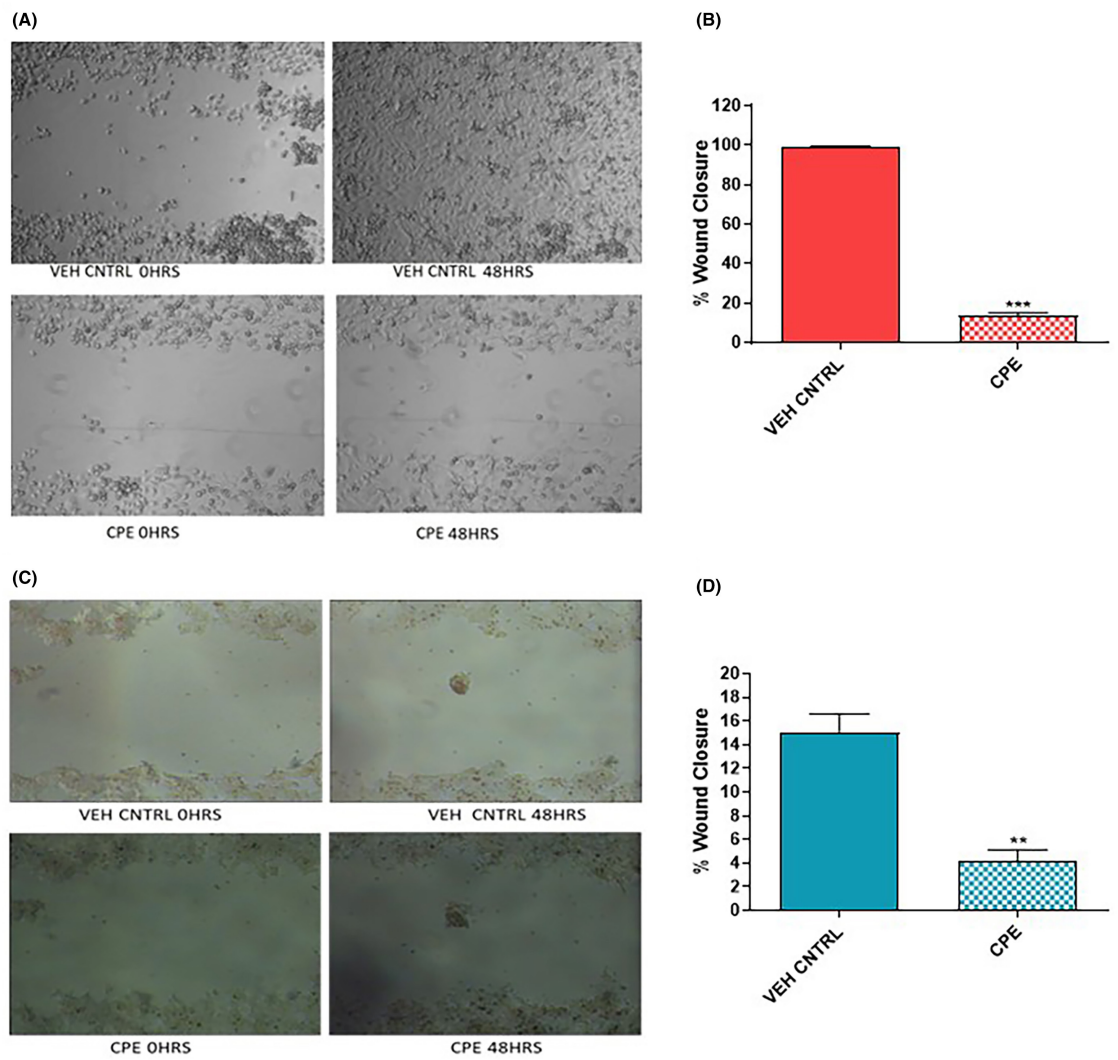
Effect of *Calotropis procera* leaf extract (CPE) on cell migration of prostate cancer cells after 48 h of treatment. Representative images showing the migration of cells in the wound area in (A) PC‐3 and (C) 22Rv1 cells. Percentage of wound closure in (B) PC‐3 and (D) 22Rv1 cells treated with CPE or vehicle control. Data are represented as mean ± SEM (*n* = 3). **p < 0.01, ***p < 0.001 compared with vehicle control.

Wound‐healing assay was performed in order to investigate the changes in the cell migration ability of PCa cells in the presence of CPE (Figure 3A,C). The percentage of wound closure in PC‐3 cells treated with CPE was 13.61%, which was significantly less (*p* < 0.001) than that of vehicle control (99.62%; Figure 3B). In 22Rv1 cells also, the cells treated with IC₅₀ value of CPE had significantly (*p* < 0.01) lower wound closure of 4.19% compared with that of vehicle control (15.08%) (Figure 3D).

### 3.5 Effect of CPE on endogenous ROS levels

**FIGURE 4:**
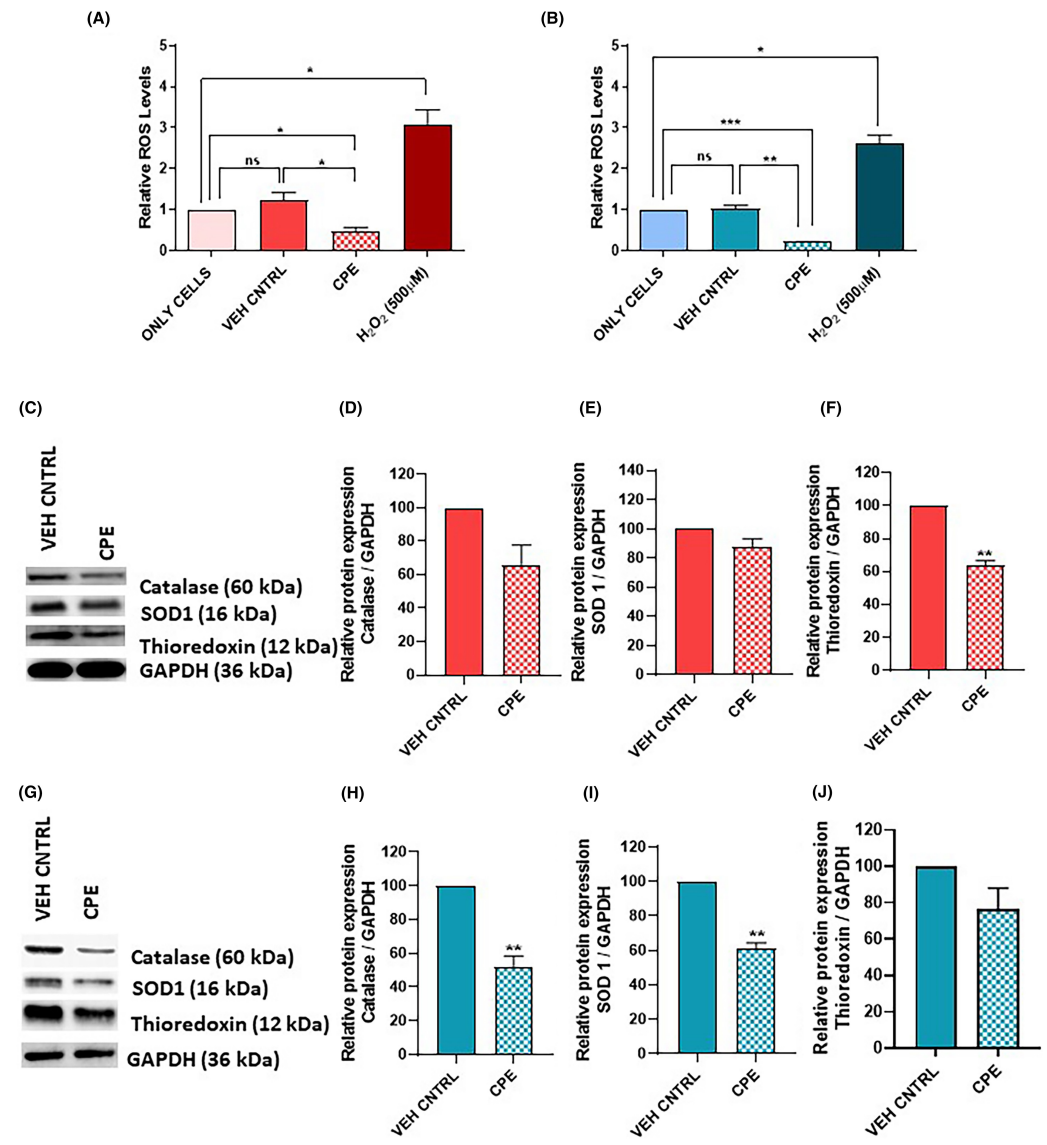
Levels of endogenous reactive oxygen species (ROS) measured by DCFDA assay and protein levels of antioxidants markers in prostate cancer cells treated with *Calotropis procera* leaf extract (CPE). Endogenous ROS levels after CPE treatment in (A) PC‐3 cells (B) 22Rv1 cells. H₂O₂ was used as positive control in this assay. (C–F) Representative western blot and densitometric analysis of antioxidant markers catalase, SOD1 and thioredoxin in PC‐3 cells. (G–J) Representative western blot and densitometric analysis of antioxidant markers catalase, SOD1 and thioredoxin in 22Rv1 cells. The data are shown as mean ± SEM (*n* = 3). ns , no significant difference; *p < 0.05, **p < 0.01, ***p < 0.001 compared with vehicle control or only cells.

DCFDA assay was performed to analyse the effect of CPE on endogenous ROS levels in PCa cells (Figure 4A,B). In both PC‐3 and 22Rv1, the ROS levels increased after treatment with H₂O₂, which was taken as positive control. Cells treated with vehicle control showed no significant change in ROS levels compared with untreated cells. However, upon treatment with IC₅₀ concentration of CPE, significant decrease in ROS levels was observed in both cell lines compared with untreated cells or compared with cells treated with vehicle control (Figure 4A,B).

### 3.6 Effect of CPE on antioxidant markers

As ROS levels decreased after treatment with CPE, the effect of CPE on protein levels of different antioxidant molecules including catalase, superoxide dismutase 1 (SOD1) and thioredoxin were analysed (Figure 4C,G). Though a reduction in catalase levels was observed after treatment of PC‐3 cells with CPE, it was not statistically significant (Figure 4D). The levels of SOD1 remained unchanged (Figure 4E) while the levels of thioredoxin decreased significantly (*p* < 0.01; Figure 4F).

Interestingly, in CPE‐treated 22Rv1 cells, protein levels of catalase and SOD1 were significantly downregulated (*p* < 0.01), while thioredoxin expression remained unchanged, as compared to vehicle control (Figure 4H–J).

### 3.7 Effect of CPE on necrosis

**FIGURE 5:**
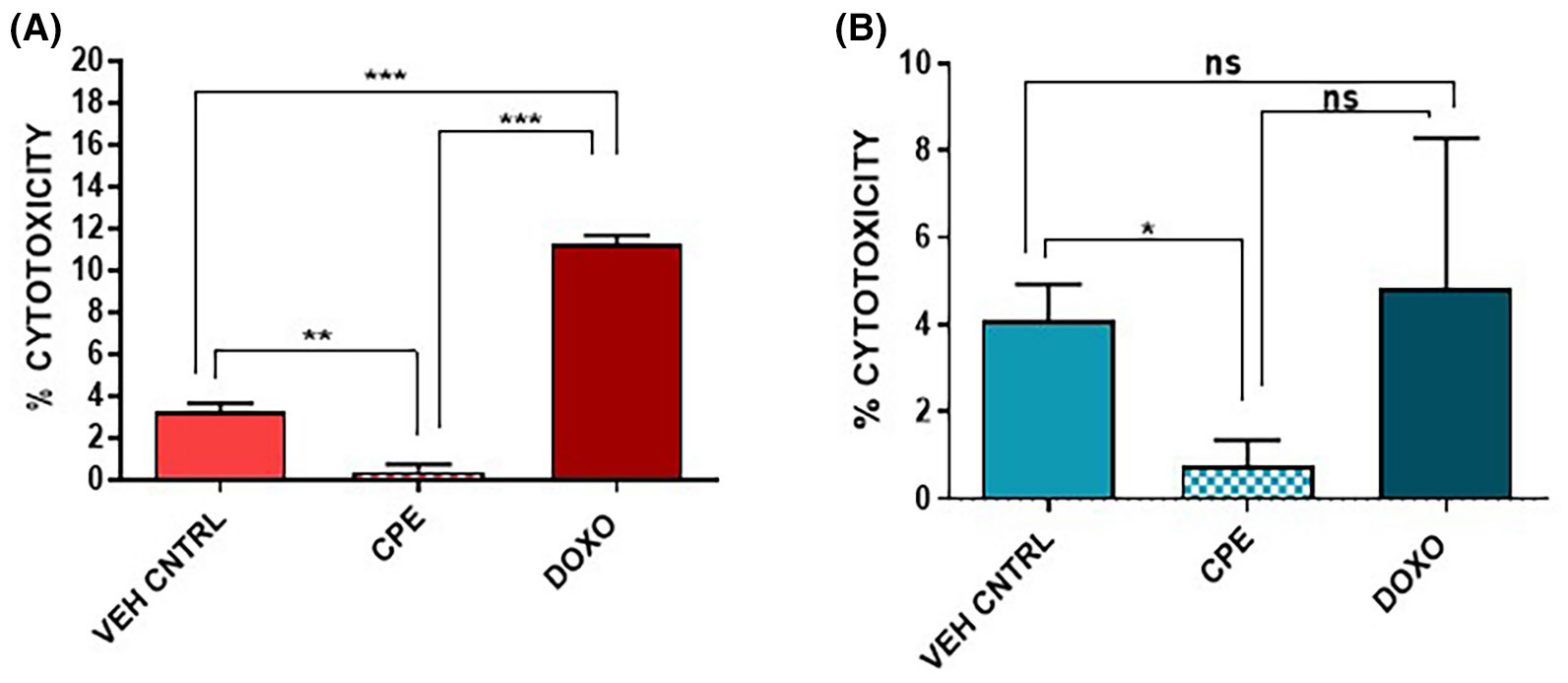
Effect of *Calotropis procera* leaf extract (CPE) on necrosis of (A) PC‐3 cells and (B) 22Rv1 cells as analysed by the lactate dehydrogenase release assay. Doxorubicin was used as positive control in this assay. The data are shown as mean ± SEM (*n* = 3). ns , no significant difference; *p < 0.05, **p < 0.01, ***p < 0.001 in CPE‐treated cells compared with vehicle control cells or doxorubicin‐treated cells.

Lactate dehydrogenase assay was performed to analyse whether CPE induces necrosis in PCa cells (Figure 5A,B). The percentage cell cytotoxicity was significantly reduced in both PC‐3 (*p* < 0.01) and 22Rv1 (*p* < 0.05) cells on treatment with CPE indicating a reduction in necrosis as compared to vehicle control (Figure 5A,B).

### 3.8 Effect of CPE on markers of autophagy

**FIGURE 6:**
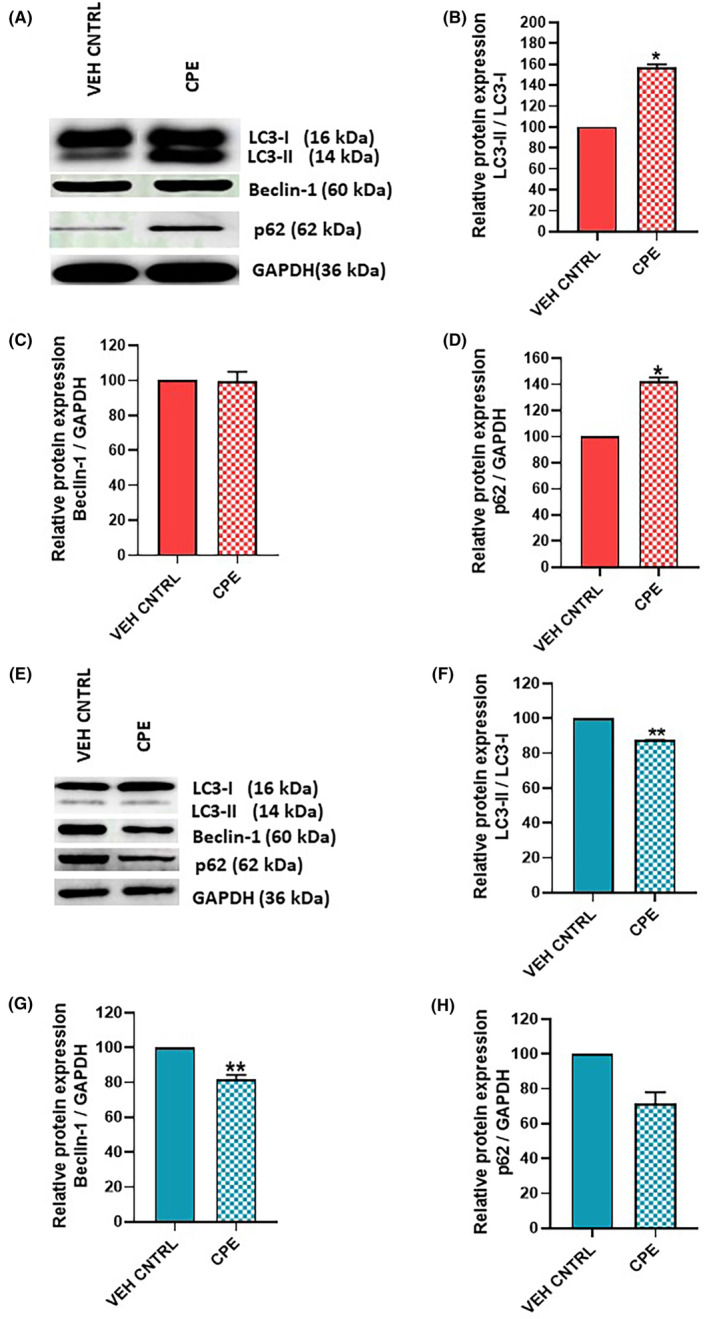
Effect of *Calotropis procera* leaf extract (CPE) on molecular markers of autophagy in PC‐3 and 22Rv1 cells. (A–D) Representative western blot and densitometric analysis of autophagy markers in PC‐3 cells. (E–H) Representative western blot and densitometric analysis of autophagy markers in 22Rv1 cells. Data are represented as mean ± SEM (*n* = 3). *p < 0.05, **p < 0.01 between vehicle control and CPE‐treated groups.

Western blotting for autophagy marker proteins, LC3B, Beclin‐1 and p62, was performed to assess whether autophagy is the mechanism involved in decreasing cell viability of PCa cells after CPE treatment (Figure 6A,E). It was observed that in PC‐3 cells, a significant increase in the ratio of LC3‐II to LC3‐I protein levels (*p* < 0.05) was observed in cells treated with CPE (Figure 6B) but no change in Beclin‐1 level was observed between vehicle control and treatment group (Figure 6C). The levels of p62 protein in PC‐3 cells were significantly upregulated (*p* < 0.05) as well (Figure 6D).

However, in 22Rv1 cells, a significant decrease in the ratio of LC3‐II to LC3‐I and Beclin‐1 (*p* < 0.01) was observed in cells treated with CPE as compared to vehicle control (Figure 6F,G). Though a decrease in p62 levels was also observed in CPE‐treated 22Rv1 cells, it was not statistically significant (Figure 6H).

### 3.9 Effect of CPE on induction of apoptosis through extrinsic pathway

**FIGURE 7:**
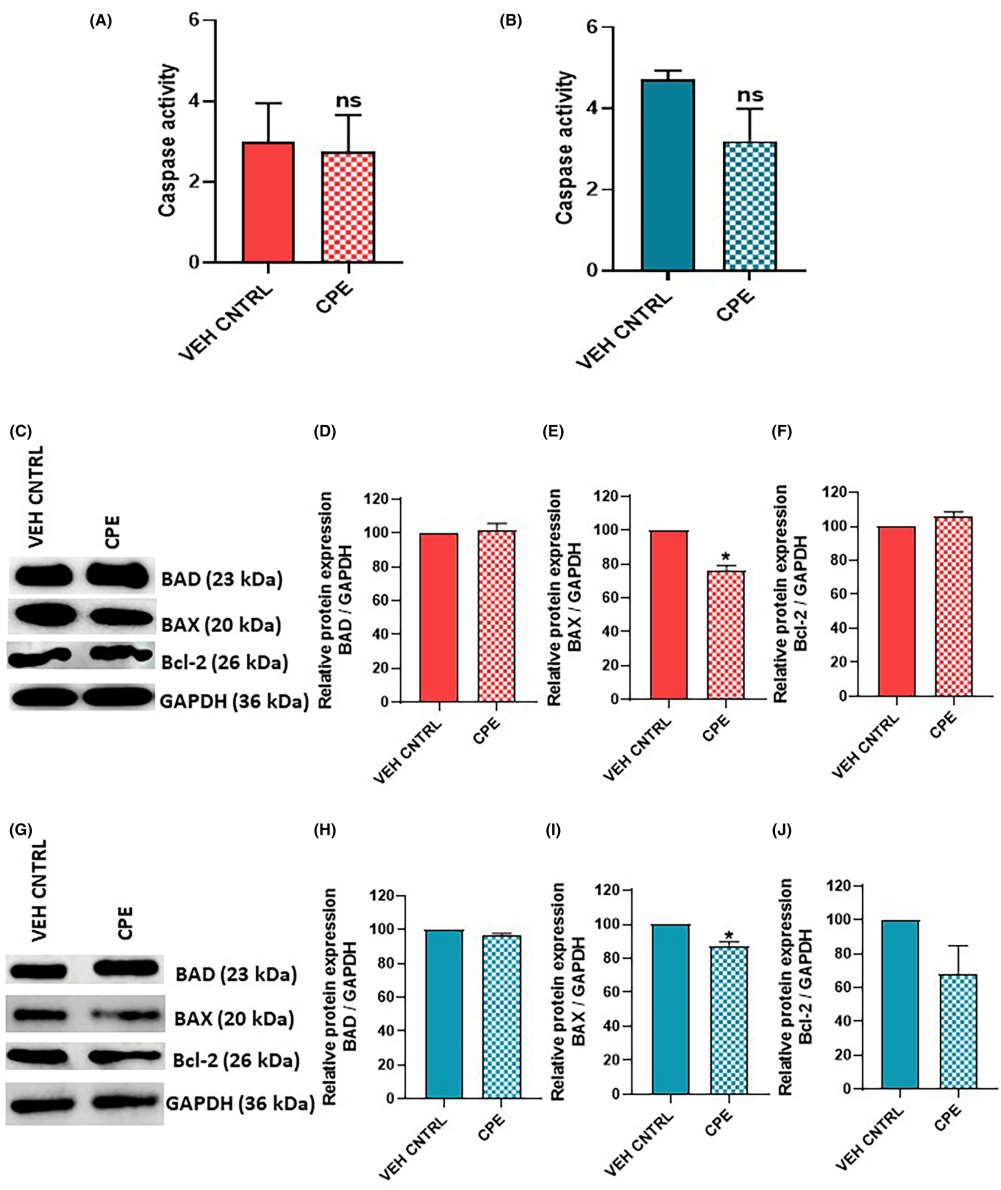
Effect of *Calotropis procera* leaf extract (CPE) on induction of apoptosis, analysed by caspase activity assay and western blot of apoptotic markers. Caspase activity after CPE treatment in (A) PC‐3 cells and (B) 22Rv1 cells. (C–F) Representative western blot and densitometric analysis of apoptotic markers BAD, BAX and Bcl‐2 in PC‐3 cells. (G–J) Representative western blot and densitometric analysis of apoptotic markers BAD, BAX and Bcl‐2 in 22Rv1 cells. The data are shown as mean ± SEM (*n* = 3). ns = no significant difference, *p < 0.05 in CPE‐treated cells compared with vehicle control cells.

Caspase activity assay was performed to analyse whether CPE reduces the cell viability of PCa cells by inducing apoptosis through extrinsic pathway. No significant change in caspase activity was observed in CPE‐treated cells as compared to vehicle control in both PC‐3 and 22Rv1 cells (Figure 7A,B).

### 3.10 Effect of CPE on apoptotic markers

In order to investigate whether decreasing cell viability by CPE was mediated through intrinsic apoptotic pathway, protein expression of apoptotic markers such as BAD, BAX and Bcl‐2 was measured in cells treated with CPE (Figure 7C,G). No change in protein level of BAD was observed while BAX level was significantly downregulated (*p* < 0.05) in CPE‐treated PC‐3 cells as compared to vehicle control (Figure 7D,E). Level of the anti‐apoptotic protein, Bcl‐2, also remained unchanged (Figure 7F).

A similar observation as PC‐3 cells was obtained in CPE‐treated 22Rv1 cells where BAD protein levels remained unchanged while a significant decrease in BAX level (*p* < 0.05) was observed (Figure 7H,I). However, a decrease in Bcl‐2 levels was also observed, though it was not statistically significant (Figure 7J).

### 3.11 LC–MS analysis of *C. procera* leaf extract

A total of 123 bioactive compounds were recognized in positive and negative ionization modes through untargeted LC–MS analysis of CPE. A list of all identified primary and secondary metabolites is given in Table 2.

**TABLE 2:** Compounds identified in *Calotropis procera* leaf extract.

S. No.	Compound	Formula	Precursor ion	Retention time (min)	Compound class	Compounds present in cell line
Compounds analysed in positive ion mode (M+H^+^)						
cp001	Safranal	C10H14O	151.10 (M+H^+^)	11.47	Cyclic terpenic aldehyde	22Rv1
cp002	Eucalyptol (cineole)	C10H18O	155.14 (M+H^+^)	13.37	Monoterpenoid	PC‐3, 22Rv1
cp003	Terpineol	C10H18O	155.14 (M+H^+^)	13.37	Monoterpenoid	22Rv1
cp004	Dihydroedulan II	C13H22O	195.17 (M+H^+^)	13.50	Organic heterobicyclic compound	–
cp005	Neryl acetone (cis‐geranylacetone)	C13H22O	195.17 (M+H^+^)	13.50	Monoterpene	PC‐3, 22Rv1
cp006	cis‐calamenene	C15H22	203.17 (M+H^+^)	15.00	Sesquiterpene	PC‐3, 22Rv1
cp007	Bicyclogermacrene	C15H24	205.19 (M+H^+^)	16.45	Sesquiterpene	22Rv1
cp008	α‐copaene	C15H24	205.19 (M+H^+^)	16.45		PC‐3, 22Rv1
cp009	α‐gurjunene	C15H24	205.19 (M+H^+^)	16.45		PC‐3, 22Rv1
cp010	cis‐thujopsene	C15H24	205.19 (M+H^+^)	16.45		PC‐3, 22Rv1
cp011	α‐humulene (alpha‐caryophyllene)	C15H24	205.19 (M+H^+^)	16.45		PC‐3, 22Rv1
cp012	β‐himachalene	C15H24	205.19 (M+H^+^)	16.45		PC‐3, 22Rv1
cp013	Germacrene D	C15H24	205.19 (M+H^+^)	16.45		PC‐3, 22Rv1
cp014	α‐selinene	C15H24	205.19 (M+H^+^)	16.45		PC‐3, 22Rv1
cp015	α‐guaiene	C15H24	205.19 (M+H^+^)	16.45		PC‐3, 22Rv1
cp016	α‐cedrene	C15H24	205.19 (M+H^+^)	16.45		PC‐3, 22Rv1
cp017	Junipene (longifolene)	C15H24	205.19 (M+H^+^)	16.45		PC‐3, 22Rv1
cp018	Trans‐caryophyllene (β‐caryophyllene)	C15H24	205.19 (M+H^+^)	16.45		PC‐3, 22Rv1
cp019	Aromadendrene	C15H24	205.19 (M+H^+^)	16.45		PC‐3, 22Rv1
cp020	α‐cadinene (α‐amorphene)	C15H24	205.19 (M+H^+^)	16.45		22Rv1
cp021	α‐cubebene	C15H24	205.19 (M+H^+^)	16.45		PC‐3, 22Rv1
cp022	α‐muurolene	C15H24	205.19 (M+H^+^)	16.45		PC‐3, 22Rv1
cp023	γ‐Cadinene	C15H24	205.19 (M+H^+^)	16.45		PC‐3, 22Rv1
cp024	β‐Muurolene	C15H24	205.19 (M+H^+^)	16.45		PC‐3, 22Rv1
cp025	Diepicedrene‐1‐oxide	C15H24O	221.18 (M+H^+^)	13.27	Sesquiterpene	22Rv1
cp026	Spathulenol	C15H24O	221.18 (M+H^+^)	14.85		22Rv1
cp027	Isoaromadendrene epoxide	C15H24O	221.18 (M+H^+^)	14.85		22Rv1
cp028	Aromadendrene oxide	C15H24O	221.18 (M+H^+^)	14.85		22Rv1
cp029	Calarene epoxide	C15H24O	221.18 (M+H^+^)	14.85		22Rv1
cp030	Cedrenol	C15H24O	221.18 (M+H^+^)	14.85		22Rv1
cp031	1,4‐trans‐1,7‐cis‐acorenone	C15H24O	221.18 (M+H^+^)	14.85		22Rv1
cp032	Cedr‐8‐en‐15‐ol	C15H24O	221.18 (M+H^+^)	14.85		22Rv1
cp033	Cedr‐8‐en‐13‐ol	C15H24O	221.18 (M+H^+^)	14.85		22Rv1
cp034	Caryophyllene oxide	C15H24O	221.18 (M+H^+^)	14.85		22Rv1
cp035	Hexahydrofarnesol	C15H32O	229.25 (M+H^+^)	13.55	Fatty alcohol	22Rv1
cp036	Cedrane‐8,13‐diol	C15H26O2	239.19 (M+H^+^)	13.27	–	PC‐3, 22Rv1
cp037	Palmitic acid	C16H32O2	257.24 (M+H^+^)	17.65	Long‐chain fatty acids	PC‐3, 22Rv1
cp038	Farnesyl acetone	C18H30O	263.23 (M+H^+^)	16.65	Terpene ketone	PC‐3, 22Rv1
cp039	9‐eicosyne	C20H38	279.30 (M+H^+^)	15.50	Aliphatic hydrocarbon	‐
cp040	Trans‐geranyl geraniol	C20H34O	291.30 (M+H^+^)	13.90	Diterpenoid	PC‐3, 22Rv1
cp041	9‐octadecenoic acid (Z)‐methyl ester	C19H36O2	297.27 (M+H^+^)	16.60	Fatty acid methyl ester	–
cp042	Phytol	C20H40O	297.31 (M+H^+^)	16.60	Terpenoid alcohol	–
cp043	Isophytol	C20H40O	297.31 (M+H^+^)	16.60		–
cp044	Methyl stearate	C19H38O2	299.29 (M+H^+^)	16.90	Fatty acid methyl esters	PC‐3, 22Rv1
cp045	Coroglaucigenin	C23H34O5	391.24 (M+H^+^)	11.85	Hydroxy steroid	PC‐3, 22Rv1
cp046	Calotropagenin	C23H32O6	405.22 (M+H^+^)	10.01	Cardenolide	PC‐3, 22Rv1
cp047	Squalene (spinacene)	C30H50	411.39 (M+H^+^)	21.20	Isoprenoid	PC‐3, 22Rv1
cp048	Stigmasterol	C29H48O	413.37 (M+H^+^)	18.45	3beta‐sterol	22Rv1
cp049	Hydroxycalotropagenin	C23H32O7	421.21 (M+H^+^)	8.01	–	PC‐3, 22Rv1
cp050	Dammaradienol	C32H52O2	427.38 (M+H^+^)	17.80	Fatty alcohols	PC‐3, 22Rv1
cp051	α‐amyrin	C30H50O	427.39 (M+H^+^)	17.80	Triterpenoid	PC‐3, 22Rv1
cp052	Multiflorenol	C30H50O	427.39 (M+H^+^)	17.85	Triterpenoid	PC‐3, 22Rv1
cp053	Lupeol (fagarasterol)	C30H50O	427.39 (M+H^+^)	17.80		PC‐3, 22Rv1
cp054	urs‐19(29)‐en‐3‐β‐ol (pyrethrol)	C30H50O	427.39 (M+H^+^)	17.80		22Rv1
cp055	β‐amyrin	C30H50O	427.39 (M+H^+^)	17.80		22Rv1
cp056	Ethyl iso‐allocholate	C26H44O5	437.32 (M+H^+^)	14.60	–	PC‐3, 22Rv1
cp057	Kaempferol hexoside	C21H20O11	449.10 (M+H^+^)	9.57	Monosaccharide derivative	PC‐3, 22Rv1
cp058	Urs‐12‐en‐24‐oic acid, 3‐oxo‐, methyl ester	C31H48O3	469.36 (M+H^+^)	20.15	–	PC‐3, 22Rv1
cp059	Urs‐19(29)‐en‐3‐yl acetate (calotropenyl acetate)	C32H52O2	469.40 (M+H^+^)	20.15	Triterpenoid	PC‐3, 22Rv1
cp060	Isorhamnetin hexoside	C22H22O12	479.11 (M+H^+^)	11.20	Glycosyloxyflavone	22Rv1
cp061	Uscharidin	C29H38O9	531.25 (M+H^+^)	11.50	Cardenolide	PC‐3, 22Rv1
cp062	Calotropin (pecilocerin A)	C29H40O9	533.27 (M+H^+^)	11.73	Cardenolide	22Rv1
cp063	Calactin (pecilocerin B)	C29H40O9	533.27 (M+H^+^)	11.73		22Rv1
cp064	Frugoside	C29H44O9	537.30 (M+H^+^)	9.61	Cardenolide	PC‐3, 22Rv1
cp065	Desglucouzarin	C29H44O9	537.30 (M+H^+^)	11.07		PC‐3, 22Rv1
cp066	12 hydroxy calactin	C29H40O10	549.26 (M+H^+^)	11.70	Cardenolide	PC‐3, 22Rv1
cp067	15 hydroxy calactin	C29H40O10	549.26 (M+H^+^)	11.70		PC‐3, 22Rv1
cp068	16 hydroxy calactin	C29H40O10	549.26 (M+H^+^)	11.70		PC‐3, 22Rv1
cp069	Calactinic acid	C29H40O10	549.26 (M+H^+^)	11.70		PC‐3, 22Rv1
cp070	Calotoxin	C29H40O10	549.26 (M+H^+^)	9.78		PC‐3, 22Rv1
cp071	Calactinic acid methyl ester I	C30H42O10	563.28 (M+H^+^)	9.64	–	PC‐3, 22Rv1
cp072	Asclepin	C31H42O10	575.28 (M+H^+^)	11.55	–	22Rv1
cp073	Voruscharin	C31H43NO8S	590.27 (M+H^+^)	16.70	Cardenolide	22Rv1
cp074	Kaempferol‐3‐O‐rutinoside (nicotiflorin)	C27H30O15	595.16 (M+H^+^)	10.45	Flavonoid glycoside	PC‐3
cp075	Kaempferol robinoside	C27H30O15	595.16 (M+H^+^)	10.45		PC‐3
cp076	15 hydroxy uscharin	C31H41NO9S	604.25 (M+H^+^)	11.75	–	PC‐3, 22Rv1
cp077	2″‐oxovoruscharin	C31H41NO9S	604.25 (M+H^+^)	11.75	Cardenolide	PC‐3, 22Rv1
cp078	Quercetin‐3‐O‐rutinoside (Rutin)	C27H30O16	611.15 (M+H^+^)	10.01	Flavonoid glycoside	PC‐3, 22Rv1
cp079	Isorhamnetin hexoside pentoside	C27H30O16	611.15 (M+H^+^)	10.01	Flavonoid glycoside	PC‐3, 22Rv1
cp080	Labriformine	C31H39NO10S	618.23 (M+H^+^)	8.63	Cardenolide	PC‐3, 22Rv1
cp081	Isorhamnetin robinoside	C28H32O16	625.17 (M+H^+^)	10.63	Flavonoid glycoside	22Rv1
cp082	Isorhamnetin‐3‐O‐rutinoside (Narcissin)	C28H32O16	625.17 (M+H^+^)	10.63		22Rv1
cp083	Calotropagenin glycoside III	–	634.27 (M+H^+^)	9.63	Calotropin derivative	–
cp084	Calotropagenin glycoside IV	–	648.28 (M+H^+^)	10.67	Calotropin derivative	22Rv1
cp085	Calotropisprocerasaponin I	C35H54O13	683.36 (M+H^+^)	17.13	–	PC‐3, 22Rv1
cp086	Uzarin	C35H54O14	699.35 (M+H^+^)	17.97	–	PC‐3, 22Rv1
Compounds analysed in negative ion mode (M‐H^+^)						
cp087	Camphor	C10H16O	151.12 (M‐H^+^)	11.47	Cyclic monoterpene Ketone	–
cp088	α‐cyclocitral	C10H16O	151.12 (M‐H^+^)	11.47	Organic oxide	PC‐3
cp089	Trans‐Pinocarveol	C10H16O	151.12 (M‐H^+^)	11.47	Bicyclic monoterpenoid	22Rv1
cp090	Verbenol (Berbenol)	C10H16O	151.12 (M‐H^+^)	11.47		22Rv1
cp091	1‐dodecene	C12H24	167.19 (M‐H^+^)	16.20	Monoterpenoid	PC‐3, 22Rv1
cp092	1,1,6‐trimethyl‐1,2‐dihydronaphthalene	C13H16	171.13 (M‐H^+^)	10.35	13C‐norisoprenoid	–
cp093	Trans‐chrysanthenol	C12H18O2	193.13 (M‐H^+^)	11.03	–	PC‐3, 22Rv1
cp094	α‐calacorene	C15H20	199.16 (M‐H^+^)	13.40	Sesquiterpenoid	PC‐3, 22Rv1
cp095	Methyl‐α‐ionone	C14H22O	205.17 (M‐H^+^)	14.23	Methyl ketone	PC‐3, 22Rv1
cp096	α‐terpinyl propionate	C13H22O2	209.16 (M‐H^+^)	11.40	p‐menthane monoterpenoid	–
cp097	Humulane‐1,6‐dien‐3‐ol	C15H26O	221.20 (M‐H^+^)	14.77	Sesquiterpene	22Rv1
cp098	Nerolidol (peruviol, penetrol)	C15H26O	221.20 (M‐H^+^)	14.77		22Rv1
cp099	Viridiflorol	C15H26O	221.20 (M‐H^+^)	14.77		22Rv1
cp100	Hinesol (agaruspirol)	C15H26O	221.20 (M‐H^+^)	14.77		22Rv1
cp101	α‐acorenol	C15H26O	221.20 (M‐H^+^)	14.77		22Rv1
cp102	Dihydro‐α‐agarofuran	C15H26O	221.20 (M‐H^+^)	14.77		22Rv1
cp103	Epishyobunol	C15H26O	221.20 (M‐H^+^)	14.77		22Rv1
cp104	Epiglobulol	C15H26O	221.20 (M‐H^+^)	14.77		22Rv1
cp105	Palustrol	C15H26O	221.20 (M‐H^+^)	14.77		22Rv1
cp106	β‐eudesmol (beta‐selinenol)	C15H26O	221.20 (M‐H^+^)	14.77		22Rv1
cp107	α‐cedrol	C15H26O	221.20 (M‐H^+^)	14.77	Sesquiterpenoid and a tertiary alcohol	22Rv1
cp108	Z‐7‐Hexadecenal	C16H30O	237.23 (M‐H^+^)	16.20	Fatty aldehyde	22Rv1
cp109	Methyl palmitate	C17H34O2	269.26 (M‐H^+^)	16.47	Fatty acid methyl Ester	22Rv1
cp110	Kaur‐16‐ene	C20H32	271.25 (M‐H^+^)	16.27	Kaurane diterpenoid	22Rv1
cp111	Bolandiol	C18H28O2	275.21 (M‐H^+^)	13.53	3beta‐hydroxy steroid	PC‐3, 22Rv1
cp112	n‐Eicosane	C20H42	281.33 (M‐H^+^)	16.60	Alkane	22Rv1
cp113	Ethyl palmitate	C18H36O2	283.27 (M‐H^+^)	16.53	Fatty acid esters	–
cp114	Quercetin	C15H10O7	301.04 (M‐H^+^)	15.40	Flavonols	22Rv1
cp115	Ethyl linoleate (mandenol)	C20H36O2	307.27 (M‐H^+^)	16.50	Linoleic acids and derivatives	–
cp116	Ethyl 9,12‐octadecadienoate	C20H36O2	307.27 (M‐H^+^)	16.50		–
cp117	Hydroxycoroglaucigenin	C23H34O6	405.24 (M‐H^+^)	9.50	Aminoquinoline	PC‐3, 22Rv1
cp118	β‐sitosterol (cupreol)	C29H50O	413.39 (M‐H^+^)	18.45	Stigmastane sterol	–
cp119	Afroside	C29H42O9	533.28 (M‐H^+^)	12.43	–	22Rv1
cp120	1‐Heptatriacontanol	C37H76O	535.59 (M‐H^+^)	20.10	–	–
cp121	Uscharin	C31H41NO8S	586.26 (M‐H^+^)	13.33	Cardenolides	PC‐3, 22Rv1
cp122	Calotropagenin glycoside II	–	600.24 (M‐H^+^)	17.80	Calotropin derivative	–
cp123	Calotropagenin glycoside I	–	634.25 (M‐H^+^)	16.75	Calotropin derivative	PC‐3, 22Rv1

Abbreviations: NI, negative ion; PI, positive ion; RT, retention time.

LC/MS analysis of PC‐3 and 22Rv1 cells treated with CPE for 24 h was performed to identify the compounds which get internalized. It was observed that from the 123 metabolites identified in the crude leaf extract, 63 metabolites were be detected in PC‐3 cells treated with CPE while they were absent in cell lysates of vehicle control (Table 2). In 22Rv1 cells, 105 metabolites were identified in the CPE‐treated cells which were absent in cell lysate of vehicle control (Table 2).

## 4 DISCUSSION

Conventional treatment methods for PCa have many side effects and hence, treatment with herbal extracts is considered an important form of alternative therapy. Thus, in this study we investigated the effect of the leaf extract of the herb, *C. procera* on PCa cell lines in vitro. Results showed that leaf extract of *C. procera* significantly decreased cell viability as well as cell division and migration capability of both androgen‐independent and androgen‐sensitive PCa cell lines. Interestingly, the androgen‐sensitive 22Rv1 cell line was more susceptible to CPE treatment as compared to androgen‐independent PC‐3 cell line as indicated by the IC_50_ values.

p27kip1 protein directly mitigates inflammation by inhibiting NF‐κB activation thus playing a key role between cell cycle and inflammation.^21^ It is also reported that loss of p27 results in increased aggressiveness of PCa.^22^ Hence, our observation that p27 increased in CPE‐treated 22Rv1 cells may explain our results showing decreased cell division and migration capability of these cells after treatment with CPE. Similarly, downregulated expression of NF‐κB in both PCa cell lines after CPE treatment is in line with another study showing that *C. procera* inhibits breast cancer proliferation by inhibiting NF‐κB activation.^23^

Elevated ROS levels maintain tumorigenicity and promote genomic instability in cancer cells. A study using various prostate cells showed that ROS generation is higher in PCa cells compared with normal prostate cells and suggested that reducing ROS production may decrease cell migration while increasing cell death.^24^ Interestingly, our data showed that CPE treatment results in significant reduction in ROS levels, which may lead to the reduced cell viability and migration capacity of the PCa cells. Moreover, another study in breast cancer has shown that *C. procera* can induce cytotoxicity by reducing ROS levels resulting in decreased cell migration and induction of apoptosis.^18^

Reduction in ROS levels is usually associated with a concomitant increase in antioxidants.^25^ Interestingly, our data showed that levels of antioxidant markers, catalase, SOD1 and thioredoxin, decreased after CPE treatment. Since CPE is known to possess significant radical scavenging activity,^16^ it can be inferred that the reduction in ROS levels observed in CPE treated PCa cells was direct and not mediated by modulation of antioxidant enzymes.

Also, SOD1 has been proposed as a novel target for anticancer therapy since it remains overexpressed in a number of cancers along with increased ROS levels in order to prevent any damage caused due to excessive ROS and maintain tumorigenesis.^26^ Similarly, another antioxidant protein thioredoxin showed upregulated expression in androgen‐independent PCa and its inhibition led to decrease in cancer growth.^27^ Thus, it can be proposed that the downregulated protein levels of SOD1 and thioredoxin could contribute to decreasing cell proliferation of PCa cells.

Cancer cells often utilize autophagy for resource reallocation as a survival strategy.^28^, ^29^ Studies have shown that in PC‐3 cells, autophagy activation is accompanied by upregulated expression of p62, LC3B and Beclin‐1 at both transcript and protein levels.^30^, ^31^, ^32^ These three proteins play a crucial role in different key steps of autophagy and, hence, were chosen for analysis. In our study, LC3‐II and p62 expression was significantly upregulated indicating autophagy induction in CPE‐treated PC‐3 cells. In contrast, CPE treatment significantly reduced the expression of Beclin‐1 and LC3‐II in 22Rv1 cells, thus showing inhibition of autophagy. There are data suggesting that depending on the cellular features of the PCa cell lines, either induction or inhibition of autophagy can result in cell survival.^28^ Studies have shown that activation of autophagy in androgen‐independent PC‐3 cells results in cell cycle arrest in G2/M phase.^33^, ^34^ However, it has been observed that autophagy acts as pro‐survival mechanism in androgen‐responsive 22Rv1 cells.^29^

Some previous studies have shown that *C. procera* extracts inhibit proliferation of human skin melanoma cells and canine mammary tumour cells via apoptosis induction.^35^, ^36^ However, our results showed that both extrinsic and intrinsic pathways of apoptosis were not activated in PCa cells by CPE treatment. Decrease in cell viability by CPE also did not involve necrosis as shown by the results of LDH Release assay.

In order to identify the compounds of *C. procera* hydroalcoholic extract which may be inhibiting cell viability of PCa cells, LC–MS analysis of the hydroalcoholic extract as well as the cells treated with CPE was performed. Majority of compounds internalized by both PCa cell lines belonged to the class of terpene derivatives, cardenolides and flavonoid glycosides. Interestingly, more of the compounds were internalized by the androgen‐responsive 22Rv1 cells with only three compounds, namely nicotiflorin, kaempferol robinoside and α‐cyclotral internalized specifically by androgen‐independent PC‐3 cells.

Terpenes like lupeol have been known to suppress tumour angiogenesis via downregulation of TNFα.^37^ Another terpene derivative hinesol has shown anti‐proliferative effect in lung cancer cells.^38^ Cardenolides like uscharin, calotropin, calactin, 2′‐oxovoruscharin, 19‐dihydrocalactin, 19‐dihydrocalotoxin, 15‐β hydroxy uscharin, asclepin and calotropagenin have been known to inhibit Na+/K+ATPase and HIF‐1α activity, which are important for tumour metastasis. Thus, these cardenolides show cytotoxic effect against breast, colon, cervical, lung and ovarian cancer.^39^, ^40^, ^41^

Flavonoids possess high antioxidant potential as their phenolic hydroxyl group can stabilize free radicals directly or they can activate antioxidant and suppress pro‐oxidant enzymes indirectly.^42^ Quercetin derivatives found in *C. procera* are known to possess high free radical scavenging activity.^43^ Also, the phytoestrogens, kaemferol and its derivatives which have anticancer effect against several androgen‐dependent cancers and thus are suitable candidates for anticancer therapy.^44^

In summary, our results show that CPE inhibits proliferation and migration of PCa cells by regulating autophagy and reducing the levels of intracellular ROS. Our results also suggest that androgen‐sensitive and androgen‐independent PCa cells respond differently to herbal formulations depicting their uniqueness which can be utilized as a potential target for PCa treatment. This necessitates the need to test the herbal extracts on different kinds of PCa cells.

The untargeted LC–MS analysis showed the presence of several metabolites in CPE, which are known to possess cytotoxic and anti‐proliferative potential against several different cancers. Many of the metabolites also have radical scavenging potential. Further studies are required to delineate the effect of each metabolite individually or in combination on the cell viability and its associated mechanisms in PCa cells. In conclusion, our findings indicate the possibility of using CPE as an alternate therapeutic agent for PCa. This could be a significant advancement in safer treatment modalities for PCa in particular and cancer in general.

## AUTHOR CONTRIBUTIONS

**Palak Singh:** Conceptualization (lead); data curation (lead); formal analysis (lead); investigation (lead); methodology (lead); software (lead); validation (lead); visualization (lead); writing – original draft (lead); writing – review and editing (equal). **Bodhana Dhole:** Formal analysis (equal); investigation (equal); project administration (equal); resources (equal); supervision (equal); visualization (equal); writing – review and editing (equal). **Jaganmoy Choudhury:** Conceptualization (supporting); project administration (supporting); software (equal); supervision (supporting); visualization (supporting). **Anannya Tuli:** Data curation (supporting); formal analysis (supporting). **Deepak Pandey:** Conceptualization (equal); methodology (equal); supervision (equal); visualization (equal). **Thirumurthy Velpandian:** Formal analysis (supporting); supervision (supporting). **Surabhi Gupta:** Conceptualization (lead); formal analysis (equal); funding acquisition (lead); investigation (equal); methodology (lead); project administration (lead); supervision (lead); validation (lead); visualization (lead); writing – review and editing (equal). **Pradeep Kumar Chaturvedi:** Conceptualization (lead); funding acquisition (lead); project administration (lead); resources (lead); supervision (lead); writing – review and editing (equal).

## CONFLICT OF INTEREST STATEMENT

The authors declare that there are no conflicts of interest.

## Data Availability

Data from this study are available from the corresponding author upon reasonable request.
